# Unifying the functional diversity in natural and cultivated soils using the overall body-mass distribution of nematodes

**DOI:** 10.1186/s12898-017-0145-9

**Published:** 2017-11-28

**Authors:** Christian Mulder, Rob Maas

**Affiliations:** National Institute for Public Health and the Environment (RIVM), Bilthoven, The Netherlands

**Keywords:** Body-mass distribution, Functional Divergence, Functional Evenness, Functional Richness, Overall diversity

## Abstract

**Background:**

Sustainable use of our soils is a key goal for environmental protection. As many ecosystem services are supported belowground at different trophic levels by nematodes, soil nematodes are expected to provide objective metrics for biological quality to integrate physical and chemical soil variables. Trait measurements of body mass carried out at the individual level can in this way be correlated with environmental properties that influence the performance of soil biota.

**Results:**

Soil samples were collected across 200 sites (4 soil types and 5 land-use types resulting in 9 combinations) during a long-term monitoring programme in the Netherlands and the functional diversity of nematode communities was investigated. Using three commonly used functional diversity indices applicable to single traits (Divergence, Evenness and Richness), a unified index of overall body-mass distribution is proposed to better illustrate the application of functional metrics as a descriptor of land use. Effects of land use and soil chemistry on the functional diversity of nematodes were demonstrated and a combination of environmental factors accounts for the low functional value of Scots Pine forest soils in comparison to the high functional value of heathland soils, whereas human factors account for the low functional and chemical values of arable fields.

**Conclusions:**

These findings show an unexpected high functional vulnerability of nematodes inhabiting clay-rich soils in comparison to sandy soils and support the notion that soil C:N ratio is a major driver of biodiversity. The higher the C:N ratio, the higher the overall diversity, as soil nematodes cope better with nutrient-poor agroecosystems under less intense fertilization. A trait-based way focusing on size distribution of nematodes is proposed to maintain environmental health by monitoring the overall diversity in soil biota, keeping agriculture and forestry sustainable.
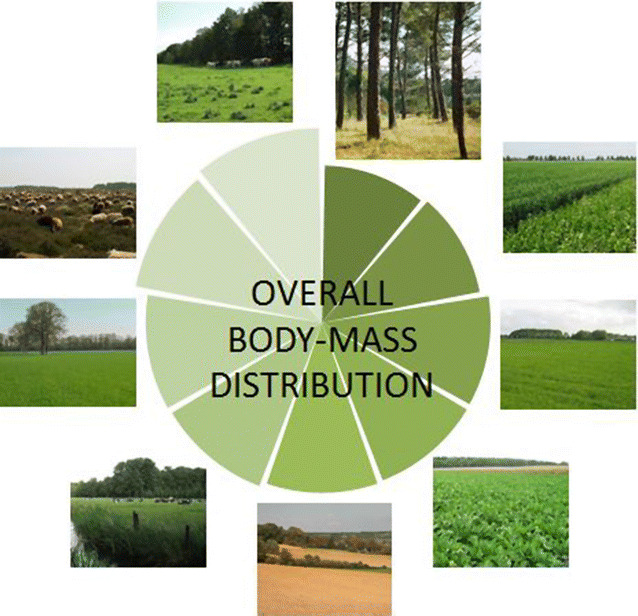

**Electronic supplementary material:**

The online version of this article (10.1186/s12898-017-0145-9) contains supplementary material, which is available to authorized users.

## Background

Preserving our thin soil is an important element in environmental policy, but the lack of a consensus on methodological criteria regarding sampling protocols and soil bioindicators is of concern. Will we ever be able to recognize good conditions for soils, and define the *stable state* of this important, non-renewable part of our ecosystems? Soil chemistry and management practices are known to impact tiny soil invertebrates. For instance, the environmental availability of key soil nutrients and the increasing liming of cultivated soils have important effects on detrital food webs and recent studies show that larger-bodied invertebrates are more sensitive to environmental changes than smaller-bodied invertebrates [1, 2]. As supporting ecosystem services are converging on soil faunal activity within multiple trophic levels, tiny invertebrates like free-living nematodes can play a major role, making them valuable proxies for belowground ecological processes [3–5].

Nematodes are among the most frequently used bioindicators due to their occurrence at multiple trophic levels of the detrital food web, their wide range of sensitivities towards external disturbances, and their easy extractability from the soil. Hence their taxonomy and life history has been widely used for functional analyses [3, 4], although body-mass investigations at the community level are almost lacking. The few existing body-mass analyses in nematology were conducted either by collecting average traits per species from the scientific literature [5] or by following cohorts in the laboratory during their entire development [6]. But, although detrital food webs are less size-structured than aquatic webs, because large, isolated nematodes can be easily attacked by smaller organisms [7], measurements of the functional diversity of soil nematodes based on their *site*-*specific body*-*mass distribution* are entirely missing.

This is rather surprising, as the size of organisms (*M*) is widely recognized as the best sole predictor in allometric models and plays a dominant role in the delivery of ecosystem services (soil heterotrophs are regarded as ecosystem engineers because they are both motors and moderators of environmental changes). Exergy (the work a system can perform when at equilibrium [8]) can be derived from the body-mass distribution of the species. In addition, a functional trait like *M*, which is so strongly correlated with the environment, can be seen as the lowest common denominator among ecological and evolutionary processes, providing a way to mechanistically understand species responses to environmental change.

This global model is likely to hold for the soil nematofauna as well, although this is not well known due the lack of knowledge on *site*-*specific body*-*mass distributions*. In 2004, Mike Kaspari already questioned for soil invertebrates: “*But why should* M *vary from place to place?*” [9] and indeed recent evidence shows that the body-mass averages of soil invertebrates strongly change from place to place according to local soil chemistry [2, 10], following the environmental-driven principles of ecological stoichiometry [1, 10] and cascading resource-consumer effects with increased land management [1, 11, 12]. However, most efforts focus on aboveground organisms and the investigation of nematodes remains uncommon. Belowground, too many studies start with soil mesofauna (mites, collembolans, enchytraeids), ending with either macrofauna or megafauna but omitting the microfauna (amoebas, ciliates, flagellates, rotifers, nematodes) and sometimes even the microflora (fungi, bacteria). For instance, in Ernest et al. [13] only one protist species was considered, and the research papers on traits in soil ecology reviewed by Pey et al. [12] address collembolans (mesofauna) or earthworms (macrofauna), but not nematodes. This means that functional trait studies remain rare in nematology, as compared to microbiology, botany or entomology.

A theoretical framework for effect and response traits was introduced by Lavorel et al. [14] and was extended by Enquist et al. [15] who make the prediction that: “*Shifts in the environment will cause shifts in the trait distribution*”. Many successful efforts have been made to predict the global distribution of functional traits for vascular plants [14–17]. Again, in the case of heterotrophs comparable site-specific efforts ranging from microflora up to macrofauna are restricted to few reference locations [18] and although valuable trait databases are being produced (David Russell, pers. comm.), most collect and provide species-specific average traits, not site-specific individual-based traits. These databases, which encompass ecosystem services and environmental information (e.g. http://www.naturalcapitalproject.org) up to biodiversity (http://www.issg.org/database, http://www.edaphobase.org), with plenty of specialized species and trait repositories like those for fishes, birds and vascular plants (http://www.fishbase.org, http://ebird.org, http://www.try-db.org, respectively), are suitable for macroecological purposes but are often unsuitable to assess local functional diversity in response to environmental drivers.

Single traits, like the individual-based body-mass values, can provide promising opportunities to derive the functional diversities of communities of autrotrophs such as algae [19] or heterotrophs such as nematodes. It is in fact likely that nematodes will reflect soil quality, but this may depend on scale. At a larger spatial scales, complex landscapes like agroecosystems are often characterized by a high level of immigration from (semi)natural habitats at the border of managed systems [2, 11, 20], explaining the high aboveground biodiversity observed in fragmented landscapes around organic farms [21, 22]. According to us, such an ecological process makes the taxon-free analysis of several site-specific distributions of one single functional trait even more relevant to explain and predict the functioning of ecosystems under pressure.

Next to agricultural pressure, soil systems may face a wide range of other stress factors, e.g. desiccation, acidification, eutrophication, climate change, and habitat fragmentation. We expect that the recognition of functional regularities at small scales must be possible in soils, as all living organisms, including nematodes, obey trait-driven power laws. Hence, we aim to assess functional diversity for 200 soil nematode assemblages, sampled in both managed and unmanaged ecosystems across the Netherlands. Our goals here are to: (1) to visualize the abiotic differences among the 200 investigated sites using multivariate analysis, (2) to correlate the body-mass distribution of the nematodes with the environmental parameters of the sites, and (3) to predict the influence of separate environmental drivers on the body-mass distribution of sampled nematodes with Generalized Linear Models.

## Data and brief methods

Functional diversity is mostly seen as the variation in multiple ecologically important traits [23]. However one single trait, body mass, already provides a huge amount of information, as energy acquisition and energy use scale with body mass *M* [1, 2]. Such a trait-based framework can then be applied to agrobiodiversity using individual measurements of soil invertebrates. The body mass of nematodes is expected to be one of the most appropriate continuous traits related at the same time to behaviour and to environmental conditions. The majority of data was compiled from pre-existing data sets contributed to the Netherlands Soil Monitoring Network [24, 25], supplemented by one databank [26] and a small number of unpublished allometric data sets. In each agroecosystem, the size (length and width) of approximately 150 identified nematodes was measured to the nearest 1 μm with an eyepiece micrometer to compute their weight (body mass) with a volumetric function.

**Fig. 1:**
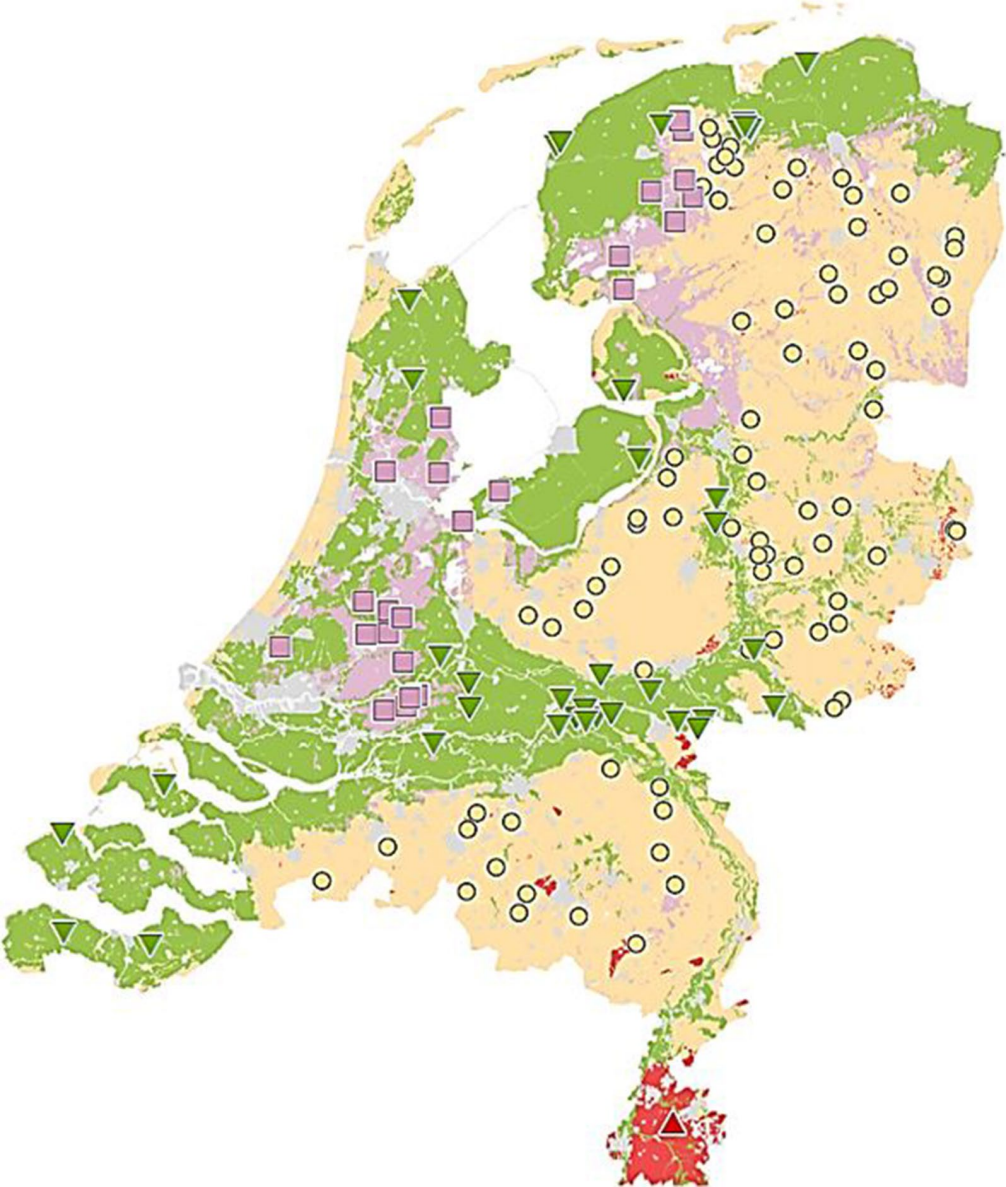
Spatial distribution of the investigated soils across the Netherlands: 118 sites were sampled on sand (circles, Podzols with creamy background), 41 on clay (inverted triangles, Fluvisols and Cambisols with greenish background), 29 on peat (squares, Histosols with purple background) and 12 on Loess (upper triangles, Luvisols with reddish background, locations too close to each other to be plotted separately). Please compare the geographical locations of the sites in this map with their Euclidean locations in Fig. 2, upper panel (a)

**Fig. 2:**
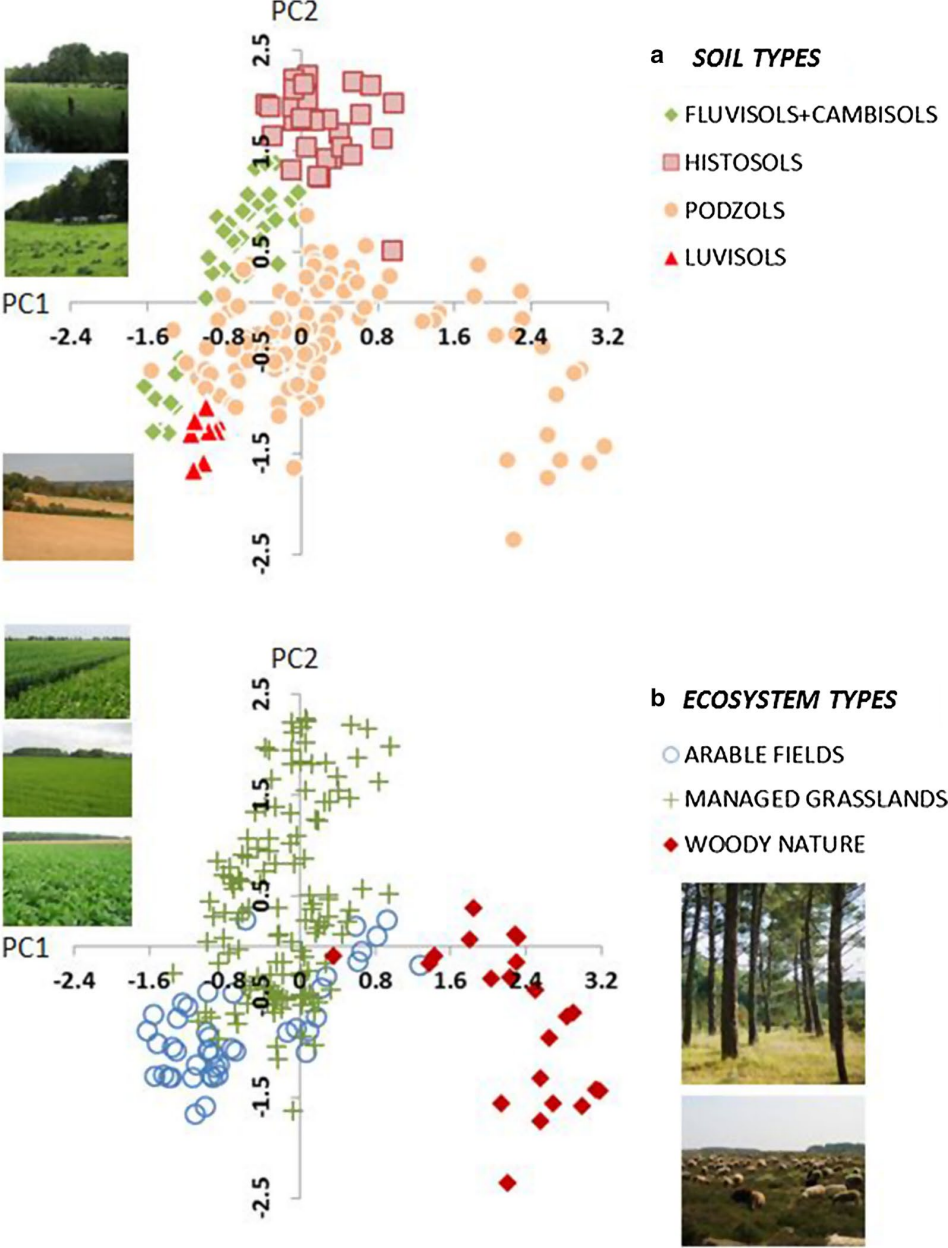
Principal component analysis (PCA) of the (log-transformed) environmental variables (soil pH, C, N, P, C:N, C:P and N:P) of the investigated sites. Rotated varimax plot(s) visualized in a multifunctional space for the first principal component by the loadings pH, C:N, N:P and C:P (52.29%) and for the second principal component by the loadings C and N (37.49%). These elemental factors are closely correlated with soil types (**a** ANOVA *F*-ratios 237.77 for C and 259.24 for N, both *p* < 0.0001), with the average P concentration of Loess and sand 2-times less than in peat, the N concentration 4-times less, and the C concentration 6-times less. The ANOVA also exhibits the expected correlation between pH and ecosystems (**b** *F*-ratio 83.21, *p* < 0.0001), as in the Netherlands woody nature is occurring on acidic soils. Photo credits: Christian Mulder, Ton Schouten, Arthur de Groot and Bert van Dijk (RIVM)

As a large number of functional diversity indices have been devised, the most widely used approach has been chosen, i.e. to apply the overall definition of functional diversity as recommended by Mason et al. [27]. This takes into account the three primary components of functional diversity (Divergence, Evenness, and Richness: full statistical explanation at the end of this paper in “Methods”—“Statistics” section). Based upon these functional components, trait-based metrics (sensu Villéger et al. [28]) were derived from all the 29,552 nematode individuals recorded in 200 soil systems (Fig. 1). The resulting components of functional diversity and the unifying average of these indices (introduced as overall body-mass distribution, hereafter *BMD*) were compared to local soil chemistry (pH, carbon, nitrogen and phosphorus contents, and molar nutrient ratios) over different environmental categories (4 soil types and 3 main management regimes: Fig. 2a, b, respectively) in an attempt to provide an indicator of soil quality and ecosystem functioning.

## Results

**Fig. 3:**
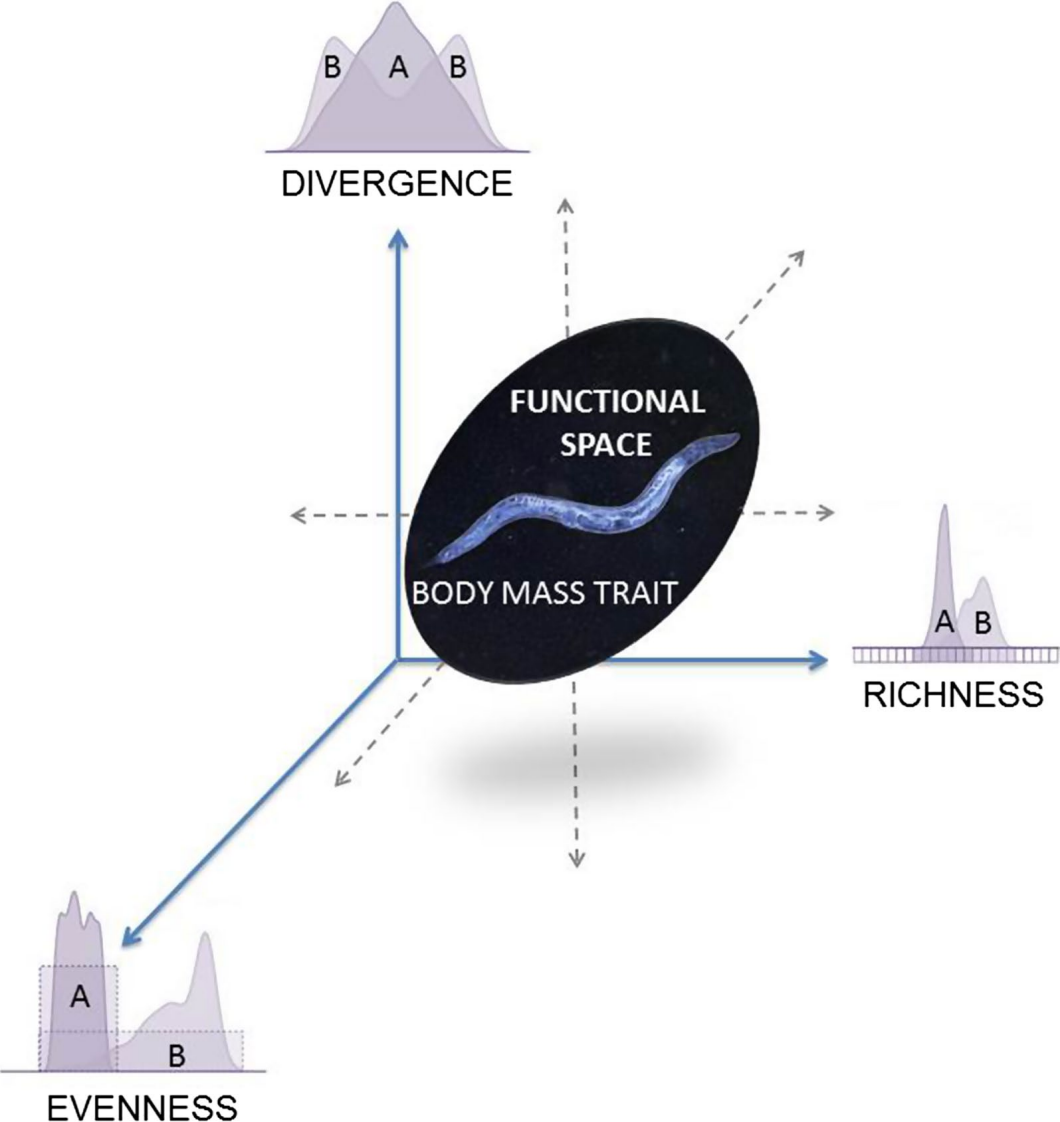
Location and variation of either species or assemblages can be visualized within a three-dimensional trait space, where in the case of species the dimensions are provided by traits and in the case of assemblages (this study) the dimensions are provided by trait-based indices. There are thus three functional components in the multidimensional space of a trait distribution (here, two nematode communities labeled as A and B for simplicity) at any given location. *Evenness* quantifies the regularity in the body-mass distribution of the individual nematodes in their functional spaces (nematofauna A or B); *Richness* quantifies the functional space occupied by the same individual nematodes with their body-mass values; *Divergence* is the degree to which the abundance in functional space of individual nematodes belonging to either nematofauna A or B is distributed towards the tails of a weight range

At the community level, we focused on three functional diversity indices: Divergence, Evenness and Richness (Fig. 3). The body-mass distribution for most taxa is far from unimodal and 89.6% of the nematode taxa exhibit a positively-skewed leptokurtic distribution. The community trait distribution closely mirrors soil environmental conditions. The trait-distribution of the nematofauna shows that these invertebrates are highly sensitive to shifts in the soil C:N ratio (Table 1) and to different management practices (always a significant factor in the Tukey’s Studentized Range test). There were significant differences between the sites in soil acidity and macronutrients. The coefficient of variation of nitrogen concentration was the highest (94.9%), followed by carbon (89.8%) and phosphorus (65.6%), while the coefficient of variation for molar ratios was the highest for C:P (134.1%), followed by N:P (87.6%) and C:N (34%). The latter result is remarkable, as despite its rather low coefficient of variation, the C:N ratio is an important driver of functional diversity metrics (Table 1).

**Table 1:** Environmental-driven functional trends (Divergence, *FD*, Evenness, *FE*, Richness, *FR*, and overall body-mass distribution, *BMD*) for positive or negative Pearson’s correlation coefficients (upper lines, italics) and significances (*n* = 200, Prob > |*r*|, lower lines) for the body-mass distribution of nematodes and soil abiotics (pH, carbon, nitrogen and phosphorus)

	*FD*	*FE*	*FR*	*BMD*
pH	*Negative*	*Negative*	Neutral	*Negative*
	*0.005*	*0.008*	0.414	*0.040*
C	Neutral	Neutral	Neutral	Neutral
	0.852	0.641	0.803	0.915
N	Neutral	Neutral	Neutral	Neutral
	0.398	0.722	0.300	0.401
P	Neutral	Neutral	Neutral	Neutral
	0.327	0.410	0.646	0.438
C:N	*Positive*	*Positive*	*Positive*	*Positive*
	*<0.0001*	*<0.0001*	*0.009*	*<0.0001*
C:P	Neutral	*Positive*	Neutral	Neutral
	0.057	*0.016*	0.322	0.395
N:P	Neutral	Neutral	*Negative*	Neutral
	0.911	0.390	*0.006*	0.289

The term ‘neutral’ was used for all the statistically not significant correlations

We found in fact that soil C:N ratio was positively related to all functional diversity indices, and hence to their average (Table 1), indicating that in soils that are nutrient-poor, either due to a lack of fertilization or due to relatively low atmospheric N-deposition, the overall size of nematodes was the lowest and the correlation of functional metrics with soil abiotics was the highest. This observation implies that nematodes in soils with lower C:N ratios are much more diverse in body size, due to a larger range and larger spacing in body sizes between coexisting soil nematodes, with unfilled bins close to highly-filled bins. Obviously this statistical finding immediately raises the question: with increasing nitrogen availability (lower C:N and higher N:P ratios), do the phenologically-larger nematodes become less abundant or smaller, or do the phenologically-smaller nematodes become more abundant or bigger? The very low Divergence values (Table 2) seem to suggest a structural homogeneity of the body-mass distribution in soil biota, but even these small changes should not to be underestimated (see next paragraph).

**Table 2:** Nematode body-mass metrics (Divergence, *FD*, Evenness, *FE*, Richness, *FR*, and Overall Body-Mass Distribution, *BMD*) for the nine investigated ecosystem types (standard deviation in brackets) ranked according to increasing *BMD* mean values: *Italics* for all indices below (*above*) the first (*third*) quartile, underline for all indices above the national average (*n* = 200)

	FD	FE	FR	BMD (%)
Scots pine forests	*0.024*	*0.625*	*0.410*	*35.3*
	(± 0.008)	(± 0.080)	(± 0.044)	(± 3.5)
Arable fields on clay	*0.024*	*0.626*	*0.432*	*36.1*
	(± 0.005)	(± 0.066)	(± 0.029)	(± 2.8)
Dairy grasslands on clay	0.032	0.629	0.476	37.9
	(± 0.010)	(± 0.052)	(± 0.055)	(± 2.3)
Arable fields on sand	0.027	0.650	0.464	38.0
	(± 0.009)	(± 0.067)	(± 0.056)	(± 2.9)
Arable fields on Loess	0.031	0.632	0.487	38.3
	(± 0.007)	(± 0.046)	(± 0.047)	(± 1.8)
Dairy grasslands on peat	0.033	0.664	0.486	39.4
	(± 0.011)	(± 0.050)	(± 0.055)	(± 2.1)
Dairy grasslands on sand	0.034	0.658	0.501	39.7
	(± 0.009)	(± 0.043)	(± 0.051)	(± 1.8)
Dry heathlands on sand	*0.049*	*0.713*	0.520	*42.7*
	(± 0.009)	(± 0.037)	(± 0.037)	(± 1.5)
Organic farms on sand	*0.048*	*0.705*	*0.561*	*43.8*
	(± 0.010)	(± 0.037)	(± 0.050)	(± 1.5)

**Fig. 4:**
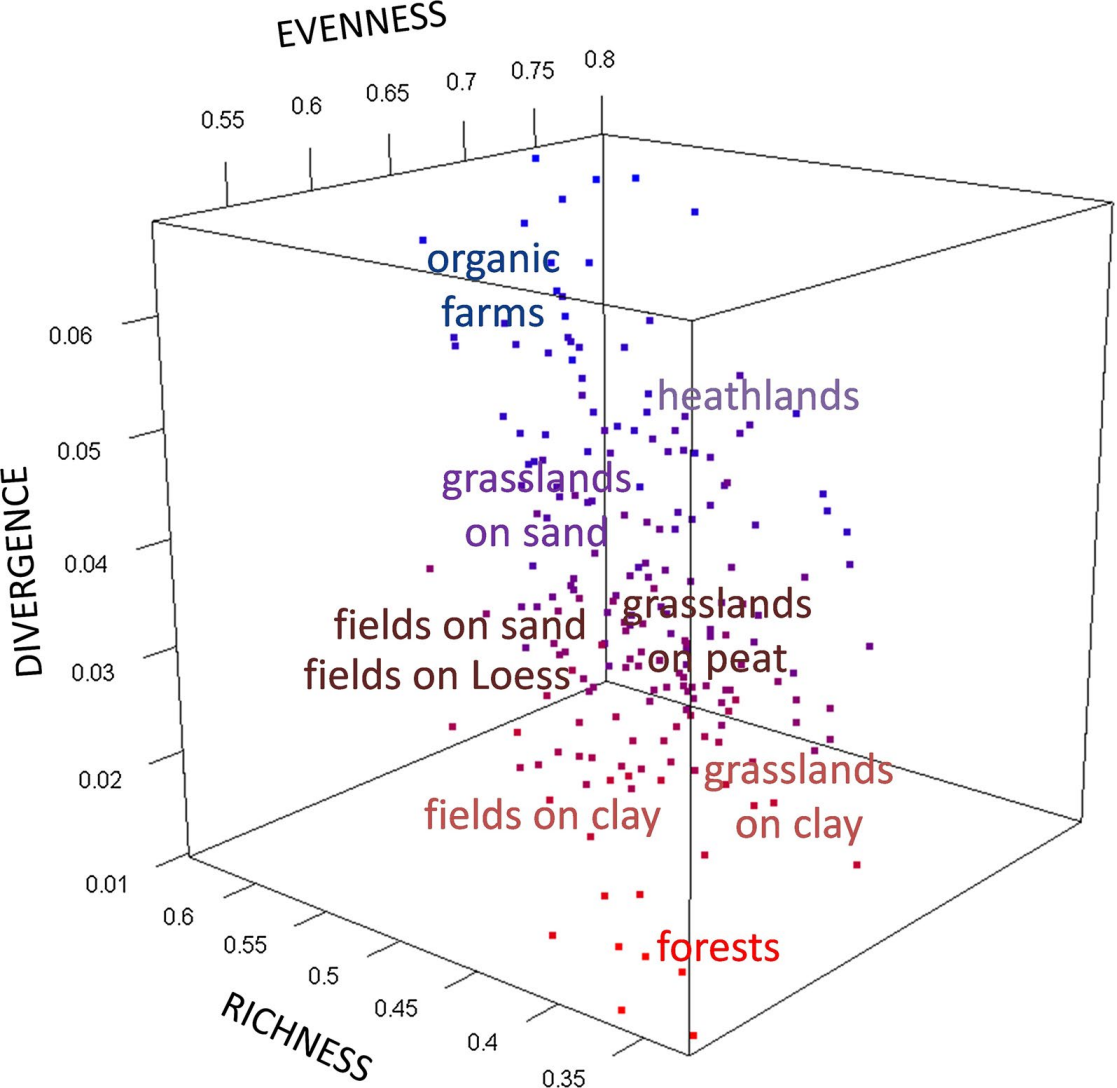
3D-scatter of the multidimensional functional space of the body-mass dispersion of the nematodes occurring in our 200 soils. The three indices (axes *x*, *y* and *z*) provide together a common currency that closely mirrors environmental filtering and hence enables to assess the overall diversity of soil systems at farm level (each single point) and at categorical level (each management practice is functionally grouped in the niche space of the nematode traits). The body-mass dispersion of nematodes in their site-specific functional space can be assessed through the trait volume of the minimum convex hull that includes all communities

A Generalized Linear Model (GLM) with Stepwise Selection (implemented forward selection technique) was used to determine the response of overall body-mass distribution (*BMD*) to the three functional diversity indices (Divergence, Evenness, and Richness). This Stepwise GLM shows that Divergence was the best single predictor of overall *BMD* (it explains 83.08% of the variation in *BMD*), followed by Evenness (11.04%) and finally by Richness (5.88%) (Fig. 4). All functional diversity indices were highly predictable by GLMs running on soil abiotics (Divergence, Richness and *BMD p* < 0.0001, Evenness *p* = 0.006).

Another stepwise GLM of the *BMD*, this time as predicted by soil abiotics (pH, C, N, P, C:N, C:P and N:P), shows soil acidity and the molar ratios as the most robust predictors, with C:N ratio having the most significant effect on *M* (*p* < 0.0001), closely followed by C:P (*p* = 0.0002), pH (*p* = 0.0215) and N:P (*p* = 0.0213). Independent GLMs show that only C:N, N:P and pH met the significance level for stepwise entry into the models forecasting Divergence (*p* < 0.0001 for both C:N and N:P and 0.0008 for pH), Evenness (*p* < 0.0001, 0.1394 and 0.0374) or Richness (*p* < 0.0001, 0.0061 and 0.0007), hence soil abiotic variables are important in structuring the entire body-mass distribution of the soil nematofauna.

There is also a major difference between the functional diversity of the nematodes in sandy vs. clay-rich soils and across natural sites, with heathland nematofauna having much higher diversity than forest nematofauna (Table 2; Additional file 1). Multiple aspects of the functional diversity of nematodes show that nematodes in clay soils are functionally less diverse than nematodes in sandy soils. This observation can be ascribed to different soil structures (less communicating water biofilm inhabited by nematodes in sandy soils) and management practices (many more pesticides on clay-rich soils according to the Dutch Central Bureau of Statistics, http://www.cbs.nl). This means that soil pore space and abundance of nematodes may play key roles in defining the overall body-mass distribution. A different soil structure does this by limiting movements and access of larger-sized predatory nematodes to their prey, as well as by supplying space acting as refuges for the resting life-stages called Dauerlarvae.

## Discussion

Using traits like body mass is an established method of great interest to numerical ecologists [29, 30]. Nematode community indices based on species-specific properties have been widely utilized to evaluate soils [3–5, 31–33], but to our knowledge this is the first study that derives nematode community indices from body mass without taking either the identity or the life-stage of single individuals into account. As these functional indices were derived from the same trait, they are correlated with each other, resulting in a constrained trait volume.

Soil faunal activity is important to understand because it is a key driver of supporting ecosystem services. Body mass of nematodes may be an appropriate continuous trait to quantify their activity and functional effects [e.g., 33–35]. Functional indices based on one easily measurable but essential soft trait like body mass are useful and cost effective because they have a solid ecological underpinning and are not influenced by differing taxonomical knowledge across laboratories. Functional diversity indices will facilitate direct comparisons across ecosystems and between countries: there are several direct applications that we are going to address separately.

### Spatial representativity

Agricultural land occupies by far the largest part of the Netherlands, with pastures being the dominant land-use type. Other forms of land use, like forests, occupy < 10% of the rural area. The major soil types are sandy soils, 50.1%, clay-rich soils, 35.7%, peaty soils, 10.6%, and only 1.6% Loess [35]. The distribution of ecosystem types mirrors the high diversity of management in the centre and east of the Netherlands (Fig. 1), with 53.9% of investigated sites on sandy soils, 23.0% on clay-rich soils, 16.3% on peat and 6.7% on Loess. Yeates [34] already stated that diversity within functional groups “*may be the key to understanding the global impacts of agricultural productions systems on nematode diversity*”. Soil biodiversity loss after land conversion has been successfully predicted [11, 36]. For instance, during land-use intensification, tillage can damage nematodes mechanically [37], disrupting soil texture and hence reducing Divergence and Evenness as shown here. Hence, the functional diversity of nematodes in clay-rich soils, where an intensive tillage regime and frequent pesticide applications are common practices, is more affected by agricultural practices than in the case of the nematofauna in sandy soils. In other words, nematodes in clay-rich soils are functionally less diverse, possibly making their detrital food webs less resilient to environmental shifts than for most agroecosystems on sand (Additional file 1). Moreover, if P is less susceptible to runoff when accumulated in larger aggregates [38], slower nematode movement in fine-textured clay would increase the isolation among local populations [39], resulting in a mismatch in the Divergence of exploited and compacted soils.

### Organic matter

Organic matter is one of the most widely investigated factors in agroecology as it influences soil water-stable aggregation during crop residue decomposition. Dutch arable fields are poor in organic matter, with 65.9% of them having less than 2% soil carbon, a threshold value for erosion. However, although it is well known that decomposition rate responds to rising temperature, nitrogen enrichment and higher atmospheric CO_2_ levels [40, 41], current models were too often unable to capture essential aspects of the impacts of nitrogen on soil carbon storage [42]. With climate change, for instance, observed effects of warming on soil C stocks are variable across sites, with either positive or negative impacts possible [43], and carbon flux is known to be rapid [44]. This variation in effects can be ascribed in part to soil priming [45–47] and contributes to one of the main pitfalls of climate scenarios: they are based on short-term responses of soil respiration and mostly do not account for responses of soil invertebrates. Chertov et al. [48] made an attempt to quantify the active contribution of soil micro- and mesofauna to the formation of organic matter, might be improved from a functional, trait-driven perspective. Assessing invertebrates active in slowly-decomposing recalcitrant organic matter, like our nematodes, quantifies carbon sequestration and may allow better estimates of soil C budgets and greenhouse gas emissions.

### Methane release

Saunois et al. [49] show that the agricultural sector in Europe is the number one contributor to the human-induced increase in global methane emission, with the majority of the annual methane emissions between 2003 and 2012 coming from the “agriculture and waste” emission category. Previous estimates showed that a rapid increase in livestock numbers is a driver of worldwide agricultural changes, with a total contribution of 15% methane by ruminants [50]. This anthropogenic trend is recognizable belowground as well, as most soil nematode taxa rapidly disappear with increasing enteric fermentation by cattle [35]. Lower nematode species richness under high livestock density may explain the higher Divergence in grazed ecosystems. Since methane production is dependent on labile C pools and as the decomposition of such labile pools in soils produces both CH_4_ and CO_2_, methane emission and manure may alter the carbon cycle. When litter reaches the soil, decomposition converts only part of the litter C into CO_2_ and most of the litter C into pools of different longevities [51]. Hence, the balance between microbes and nematodes, specifically between rapidly-decomposing bacterial cells and bacterial grazers and slowly-decomposing fungal remains and fungal grazers [52, 53], a balance so relevant for many beneficial species that outweigh pests and pathogens, is likely to be altered with high addition of cattle manure.

### Nutrient turnover

Our results show clear differences in overall functional diversity of soil nematodes depending on the land use type. Diversity was highest on organic farms and heathlands grazed by sheep (both ecosystem types with only organic fertilizers sharing the highest functional quality), followed by all other agroecosystems (each of them with either organic and mineral fertilizers or only with mineral fertilizers as for arable fields) and finally Scots Pine forest (no addition of nutrients at all and the lowest functional quality of the nematofauna: Additional file 1). Chertov [54] assumes that nutrient turnover and C:N:P stoichiometric relationships can be mediated by soil biota [53, 55], for instance by their necromass. This will be particularly true for phosphorus. As biologically-available P is thought to increase with the soil pH [1], water balance and liming, reflecting a globally challenging Ca^2+^ supply rate [56], might enhance the numerical density of soil nematodes.

### Quality assessment

In the short term, we might expect a reliable taxon-free automation in the trait estimation of soil nematodes with flow-cytometric analysis, but meanwhile the trait estimation has to been done by light microscopy. Traits can be used to evaluate ecosystems according to their ecological potential. The three resulting functional indices can be expressed in percentages and their multi-layered average (*BMD*) clearly shows that in the Netherlands (i) organic farming is a sustainable land use, (ii) arable fields are exploited soil systems, (iii) productive agroecosystems on clay-rich soils are of lower functional diversity, and (iv) natural sites on the same soil type (acidic sand) can behave in opposite ways according to the tree canopy, with the overall functional diversity of nematodes in open canopy heath lands much higher than the overall functional diversity of close canopy forests. Functional metrics provides the tool to assess the quality of soil functions and enable to investigate and manage properly the Pandora’s Box beneath us all.

## Conclusions

According to West and Brown [57], scaling of body mass is a potent tool in any physical system, from molecules up to forests. For them, the starting point for allometric analysis was to recognize that complex structures require close integration [57]. This makes a more widespread use of body-mass distribution almost imperative for revealing some trends in soil functions, like nutrient cycling. Negative environmental developments, such as rapid human growth, increasing land use intensification and climate change, support the scenario that some soil systems might become unsustainable. It is therefore surprising that a comparable attempt to quantify functional components for soil nematodes has not been done yet, as the trait ‘body mass’ underpins the growth and dynamics—and hence the sustainability—of living organisms and the systems they belong to [57, 58]. For instance, functional trait theory has been applied in management decision-making processes and as a means of preserving some urban services in twenty-first century cityscapes, as exemplified in future planning schemes [cf. 58]. Notwithstanding a high diversity of free-living nematodes, their individual body-mass values provide precious information on the complex structure of soil systems. Hence, from a trait-based perspective our unified evidence might have comparable implications for decision-making processes on the surveillance and forecasting of effects due to agricultural intensification and global changes. The most remarkable results are that it is not the nutrient concentration that matters, but the ratios between soil macronutrients, and that the functional resilience of clay-rich soils is more endangered by agricultural practices than the functional resilience of managed sandy soils. Intensive management practices at the farm level will have global implications as well. Aside from the ongoing concern about declining biodiversity and the primary losses of crop landraces, we are facing a new kind of genetic erosion, this time of soil functions, a loss that must be addressed in situ with a much more sustainable agriculture.

## Methods

### Study area

Soil biota from 200 sites across the Netherlands were sampled during the period 2004–2009 (Fig. 1). Investigated ecosystems were either cultivated (organic farms, dairy grasslands, or arable fields) or unmanaged (*Pinus sylvestris* forests or *Calluna vulgaris* heathlands). Agroecosystems can be ranked qualitatively according to recent management regime into three categories: low-pressure (28 organic grassland farms), middle-pressure (106 dairy grasslands, mostly conventional), and high-pressure (44 arable fields). Due to the lack of agroforestry, Scots Pine forests (*n* = 12) can be regarded as no-pressure lands and are, like dry heathlands (*n* = 10), typical examples of protected nature areas in the Netherlands. The data set used here contains the following ecosystem types: arable fields on clay, arable fields on sand, arable fields on Loess, dairy grasslands on clay, dairy grasslands on peat, dairy grasslands on sand, dry heathlands on sand, organic farms on sand, and pine forests on sand.

All arable fields were winter farms, i.e. lands not cultivated or grazed at the time of sampling, including multi-cropping, intercropping, crop rotation, and alley cropping. Organic and biodynamic farming techniques were used on certified organic farms, often together with agronomic practices to enhance nitrogen fixation by clovers. Compost and farmyard manure were used for fertilization in organic farms, and no biocides were employed, in contrast to other management regimes. There biocides were used, as in conventional farms, where mineral fertilizers were used to compensate for the smaller amount of farmyard manure, and in (semi)intensive farms, where both organic and mineral fertilizers were used. Fertilizer use information was gathered through farmer interviews during the field sampling, and supplemented by monitoring data.

Relationships between soil nematode communities and the relative soil pH values (pH in H_2_O) and molar ratios carbon to nitrogen (C:N), carbon to phosphorus (C:P), and nitrogen to phosphorus (N:P) were investigated. The pH value was obtained using a de-ionized 4:1 water:soil vol/vol ratio, the C content was derived from the fresh soil organic matter after oven-combustion at 550 °C using pedotransfer factors, the N content was determined by a titrimetric method after Kjeldahl destruction and the P content by automated ion analyser after sample digestion.

In each agroecosystem, one bulk sample was produced from 320 cores (ø 2.3 × 10 cm) randomly distributed across the investigated site. The bulk of 500 g soil was kept in glass containers and stored at 4 °C prior to extraction. The nematode extraction from 100 g of soil was performed using the Oostenbrink method (a standard technique widely accepted in nematology for morphological and taxonomical purposes, even for molecular analysis; see [59, 60] for a methodological discussion). All the individual nematodes within two clean 10 ml water suspensions were screened and approximately 150 randomly-chosen specimens per site were identified under a light microscope (Table 3). All these 29,552 specimens were measured to the nearest 1 μm with an eyepiece micrometer for the traits: individual length, individual width, and individual fresh weight [26]. The latter fresh weight was derived at the individual level with a volumetric function based on the cylindrical morphology of elongate nematodes, and converted to dry body mass using a weight ratio of 0.20 [61]. For each sampling site, the trait distribution was derived from site-specific individual masses by discretizing them into equal mass bins and estimating the total mass of each class. Individuals were allocated to mass bins of width 0.0029, estimated as $h = \left( {3.5 \times SD} \right)/\sqrt n$, where *h* is the class width and *n* the total number of observations [62].

**Table 3:** List of the investigated nematode taxa

*Achromadora* sp.	*Dorylaimoides* sp.	*Plectus* sp.
*Acrobeles* sp.	*Ecumenicus monohystera*	*P. acuminatus*
*A. ciliatus*	*Epidorylaimus* sp.	*P. armatus*
*A. complexus*	*E. agilis*	*P. cirratus*
*A. mariannae*	*E. lugdunensis*	*P. elongatus*
*Acrobeloides* sp.	*Eucephalobus* sp.	*P. longicaudatus*
*A. nanus*	*E. mucronatus*	*P. parietinus*
*Aglenchus* sp.	*E. oxyuroides*	*P. parvus*
*A. agricola*	*E. striatus*	*P. pusillus*
*Alaimus* sp.	*Eudorylaimus* sp.	*P. rhizophilus*
*A. meyli*	*E. centrocercus*	*Pleurotylenchus* sp.
*A. primitivus*	*Eumonhystera* sp.	*Pratylenchus* sp.
*Amphidelus* sp.	*E. vulgaris*	*P. crenatus*
*Amplimerlinius* sp.	*Filenchus* sp.	*P. fallax*
*A. caroli*	*F. vulgaris*	*P. neglectus*
*A. icarus*	*Helicotylenchus* sp.	*P. penetrans*
*Anaplectus* sp.	*H. pseudorobustus*	*P. thornei*
*A. grandepapillatus*	*H. varicaudatus*	*P. vulnus*
*A. granulosus*	*Hemicycliophora* sp.	*Prionchulus punctatus*
*Anatonchus* sp.	*Heterocephalobus* sp.	*Prismatolaimus* sp.
*A. tridentatus*	*H. elongatus*	*P. dolichurus*
*Aphelenchoides* sp.	*Heterodera* sp.	*P. intermedius*
*A. bicaudatus*	Hoplolaimidae	*Prodorylaimus* sp.
*A. blastophthorus*	*Longidorus* sp.	*P. acris*
*A. composticola*	*L. elongatus*	*Psilenchus*
*Aphelenchus* sp.	*Malenchus* sp.	*P. hilarulus*
*A. avenae*	*M. acarayensis*	*Pungentus* sp.
*Aporcelaimellus* sp.	*M. andrassyi*	*P. alpinus*
*A. obtusicaudatus*	*M. bryophilus*	*P. silvestris*
*A. paraobtusicaudatus*	*Meloidogyne* sp.	Qudsianematidae
*A. simplex*	*M. chitwoodi*	*Quinisulcius* sp.
*Bastiania* sp.	*M. hapla*	Rhabditidae
*Bitylenchus dubius*	*M. naasi*	*Rotylenchus* sp.
*B. maximus*	*Mesodorylaimus* sp.	*R. buxophilus*
*Boleodorus thylactus*	*M. aberrans*	*R. goodeyi*
*Bunonema* sp.	*M. bastiani*	*R. robustus*
*B. reticulatum*	*M. derni*	*Seinura* sp.
Cephalobidae	*M. spengelii*	*Teratocephalus* sp.
*Cephalobus* sp.	*Metateratocephalus* sp.	*T. costatus*
*C. persegnis*	*M. crassidens*	*T. tenuis*
*Cervidellus* sp.	Monhysteridae	*Theristus agilis*
*C. serratus*	Mononchidae	*Thonus* sp.
*C. vexilliger*	*Mononchus* sp.	*T. circulifer*
*Chiloplacus* sp.	*M. aquaticus*	Thornenematidae
*C. bisexualis*	*M. truncatus*	*Thornia propinqua*
Chromadoridae	*Mylonchulus* sp.	*Trichodorus* sp.
*Chronogaster* sp.	Neodiplogasteridae	*T. primitivus*
*Clarkus* sp.	Nordiidae	*T. similis*
*C. papillatus*	*Odontolaimus chlorurus*	*Tripyla* sp.
*Coslenchus* sp.	*Panagrolaimus* sp.	*T. cornuta*
*C. costatus*	*P. detritophagus*	*T. filicaudata*
Criconematidae	*P. rigidus*	*Trophurus* sp.
*Cuticularia* sp.	*Paramphidelus* sp.	Tylenchidae
Dauerlarvae	*P. hortensis*	*Tylencholaimus* sp.
*Diphtherophora* sp.	*Paratrichodorus* sp.	*T. crassus*
*D. obesa*	*P. pachydermus*	*Tylenchorhynchus* sp.
*Diploscapter coronatus*	*P. teres*	*T. striatus*
*Discolaimus* sp.	*Paratylenchus* sp.	*Tylenchus* sp.
*Ditylenchus* sp.	*P. bukowinensis*	*T. arcuatus*
*D. myceliophagus*	*P. microdorus*	*T. elegans*
Dolichodoridae	*P. nanus*	*Tylolaimophorus typicus*
*Dolichorhynchus* sp.	*P. projectus*	*Wilsonema* sp.
*D. lamelliferus*	*P. tateae*	*W. otophorum*
*Dorydorella bryophila*		*Xiphinema* sp.
*Dorylaimellus* sp.		*X. diversicaudatum*

### Verification

Every specimen from a site-specific survey was compared to pre-existing records for other agroecosystems, i.e. comparable soil types and ecosystem types, to insure that errors had not been made in the measurements of nematode traits. Soil abiotic predictors were compared with existing GIS values and data were periodically spot checked by people using the database who found oddities or outliers. Questions regarding particular records were answered by referring to the original datasheets. Greatest care was taken to detect incorrect taxonomical identification and wrong body size measurements. During the entire process, random checking of taxa and traits (from misspelling to identification) was performed on a regular basis. Dubious taxa recorded only once as single specimen, like the marine *Daptonema*, were removed from our data set (Table 3). In addition, in EXCEL 2007 the function “Data: Remove Duplicates” was applied to remove double entries. Corrections were made based on original datasheets or notes. Information outside the norms (e.g. stake numbers that do not exist, undocumented 5-digit species codes, body sizes (body masses) either too short (small) or too long (large) for the identified taxon) was systematically checked and compared to the original data forms filled in at the Dutch Agriculture and Horticulture Laboratory (scanned as PDF files) and all ACCESS XP and EXCEL 2007 datasheets.

### Statistics

As functional diversity cannot be summarized by one single number, even if computed for a single functional trait, a framework composed of three independent components (Divergence, Evenness, and Richness) has become widely used [27, 28]. These three separate functional diversity indices were computed in R (version 3.3.3, cran.xl-mirror.nl) as follows:

- *Functional Divergence of trait-level distribution* (*FD*) quantifies how much of a body-mass distribution in a functional space maximises the divergence among traits in assemblage *i* [27, 63]. The *FD* in an assemblage (Fig. 3) is based on an abundance-weighted sum of squares analogous to a log-transformed variance with the formula:$FD = \frac{2}{\pi }\arctan (5V)$, with $V = \sum\nolimits_{i = 1}^{n} {\left[ {(\ln C_{i} - \overline{\ln C} )^{2} \times } \right.} \left. {A_{i} } \right]$ where *C*_*i*_ is the character value of the category body size for the *i*th body-mass class, *A*_*i*_ the proportional abundance of the *i*th body-mass value for the (dry weight) classes in the trait distribution of nematodes, and $\overline{\ln C}$ the abundance-weighted mean of the natural logarithm of body-mass values for the categorical classes [27]. This index is constrained by the factor ^2^⁄_π_ between 0 and 1, with 1 for a complete functional divergence.
- *Functional Evenness of trait-level distribution* (*FE*) describes how the extent to which abundance is equally distributed in the functional space in assemblage *i* [63, 64]. Several evenness indices have been proposed [64–66], like the recently introduced “Trait Even Distribution” [67]. Here, we have selected the most established functional diversity index, where *FE* represents the degree to which the body mass of the nematofauna is evenly distributed along the mass spectrum (Fig. 3). Evenness was applied to the total mass in each bin with the formula:
  $$FE = 1 - \frac{2}{\pi }\arctan \left[ {\sum\limits_{{s_{1} = 1}}^{n} {{{\left( {\ln \left( {x_{{s_{1} }} } \right) - \sum\limits_{{s_{2} = 1}}^{n} {\left( {{{\ln \left( {x_{{s_{2} }} } \right)} \mathord{\left/ {\vphantom {{\ln \left( {x_{{s_{2} }} } \right)} n}} \right. \kern-0pt} n}} \right)} } \right)^{2} } \mathord{\left/ {\vphantom {{\left( {\ln \left( {x_{{s_{1} }} } \right) - \sum\limits_{{s_{2} = 1}}^{n} {\left( {{{\ln \left( {x_{{s_{2} }} } \right)} \mathord{\left/ {\vphantom {{\ln \left( {x_{{s_{2} }} } \right)} n}} \right. \kern-0pt} n}} \right)} } \right)^{2} } n}} \right. \kern-0pt} n}} } \right]$$
  where *n* is the total number of mass bins and *x*_*i*_ the total mass of the *i*th mass bin. Also *FE* has the advantage that it varies between 0 and 1 (with 1 for a complete functional evenness) and to discriminate assemblages with statistical robustness [27, 64–66].
- *Functional Richness* of trait-level distribution (*FR*) represents the functional space *FS*_*i*_ filled by any nematode assemblage *i* (Fig. 3) with the formula:
  $$FR \, = \, \left( {\frac{FSi}{R}} \right)$$
  where *R* is the absolute range of the functional trait [27, 63]. For each of the sites, *FR* was calculated as the ratio (0 < *FR* < 1) between the mass spectrum filled by the nematofauna within its assemblage and the cumulative mass spectrum calculated over all 29,552 records [26], with 1 for a completely filled range. Hence, *FR* was calculated as a one-dimensional index for the body-mass distribution of all species [68] and we did not calculate richness using a multidimensional index estimating the minimal convex hull containing all species in one functional space [19, 28].
- *Overall body*-*mass distribution* (*BMD*) is proposed to provide a single measure of nematode functional diversity. We calculated it as a dimensionless percentage of the average of the three indices, using the formula:
  $$BMD(\% ) = \left\lceil {\overline{FD + FE + FR} } \right\rceil \times 100$$
  where each component (*FD*, *FE* and *FR*) represents one layer that can be plotted along one axis of Fig. 3. Building an optimal functional space is a critical modelling step [69] but such an additional standardisation in order to keep the functional diversity indices homogeneous allows us to put equal weight on each functional component (Additional file 1).
- *Generalized Linear Models* (*GLMs*) were fitted to the data by maximum likelihood estimation with stepwise regressions for *BMD* as function of the other three functional components *FI*, with Soil Type and Ecosystem Type as CLASS variables. All *GLMs* were done in SAS 9.4 (PROC GENMOD). First, the general form of the *GLM* was *BMD* = α + β_1_ *FI*_1_ + β_2_ *FI*_2_ + β_3_ *FI*_3_ (CLASS = Soil Type, Ecosystem Type), with *BMD* as the estimated overall body-mass distribution, *FI*_n_ each computed functional diversity index (*FD*, *FE* and *FR*, respectively), β_n_ the linear coefficient for the indices and α is the intercept. Second, for all indices (*BMD*, *FD*, *FE* and *FR*, generalized as *I*_*F*_), a comparable *GLM* was computed as function of soil abiotics. The general form of the model is $I_{(BMD,FD,FR,FR)} \propto$ α + β_1_ pH + β_2_C + β_3_N + β_4_P + β_5_(C:N) + β_6_(C:P) + β_7_(N:P), again with Soil Type and Ecosystem Type as CLASS variables. Some levels of interaction involving classification variables (nature on sand but not on clay) are not represented and GENMOD does not include missing levels. We used the same CLASS variables in one-way analysis of variance (PROC ANOVA statement).
- *Principal component analysis* (*PCA*) of the aforementioned soil predictors pH, C, N, P, C:N, C:P and N:P were log-transformed and their principal components were visualized in a multifunctional space in rotated varimax plots for all the 200 investigated sites.

### Additional file

**Additional file 1.** Graphical abstract. The nematological data built in a series of layers can be merged together into one single, dimensionless index, representing the overall soil functional diversity for each of the ecosystem types. Image credit: Christian Mulder.

## Abbreviations

BMD: body-mass distribution
C: soil carbon
FD: functional divergence
FE: functional evenness
FR: functional richness
N: total nitrogen
P: total phosphorus
