# Supplementary figures and images for: Unifying the functional diversity in natural and cultivated soils using the overall body-mass distribution of nematodes
Source: BMC Ecol. 2017 Nov 28;17:36. doi: 10.1186/s12898-017-0145-9 (PMC5706308; doi:10.1186/s12898-017-0145-9)

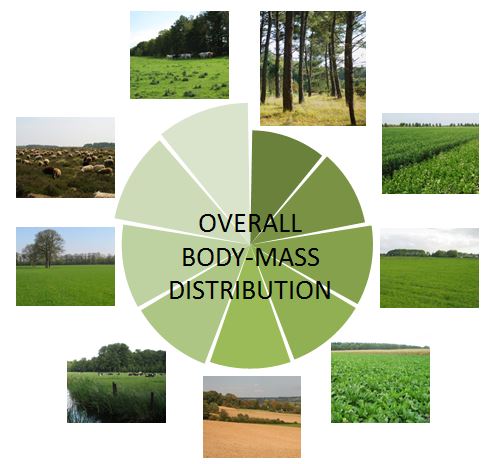

Supplement: Supplementary file 1 — Additional file 1. Graphical abstract. The nematological data built in a series of layers can be merged together into one single, dimensionless index, representing the overall soil functional diversity for each of the ecosystem types. Image credit: Christian Mulder. [file 12898_2017_145_MOESM1_ESM.jpg]
